# Familial Adrenocortical Carcinoma in Association With Lynch Syndrome

**DOI:** 10.1210/jc.2016-1460

**Published:** 2016-05-04

**Authors:** Benjamin G. Challis, Narayanan Kandasamy, Andrew S. Powlson, Olympia Koulouri, Anand Kumar Annamalai, Lisa Happerfield, Alison J. Marker, Mark J. Arends, Serena Nik-Zainal, Mark Gurnell

**Affiliations:** Metabolic Research Laboratories (B.G.C., N.K., A.S.P., O.K., A.K.A., M.G.), Wellcome Trust-MRC Institute of Metabolic Science, and Departments of Histopathology (L.H., A.J.M.) and Medical Genetics (S.N.-Z.), University of Cambridge and National Institute for Health Research Cambridge Biomedical Research Centre, Addenbrooke's Hospital, Cambridge, UK CB2 0QQ; Division of Pathology (M.J.A.), University of Edinburgh, Edinburgh, UK

## Abstract

**Context:**

Adrenocortical carcinoma (ACC) is a rare endocrine malignancy with a poor prognosis. Although the majority of childhood ACC arises in the context of inherited cancer susceptibility syndromes, it remains less clear whether a hereditary tumor predisposition exists for the development of ACC in adults. Here, we report the first occurrence of familial ACC in a kindred with Lynch syndrome resulting from a pathogenic germline *MSH2* mutation.

**Case:**

A 54-year-old female with a history of ovarian and colorectal malignancy was found to have an ACC. A detailed family history revealed her mother had died of ACC and her sister had previously been diagnosed with endometrial and colorectal cancers. A unifying diagnosis of Lynch syndrome was considered, and immunohistochemical analyses demonstrated loss of MSH2 and MSH6 expression in both AACs (proband and her mother) and in the endometrial carcinoma of her sister. Subsequent genetic screening confirmed the presence of a germline *MSH2* mutation (resulting in deletions of exons 1–3) in the proband and her sister.

**Conclusion:**

Our findings provide strong support for the recent proposal that ACC should be considered a Lynch syndrome-associated tumor and included in the Amsterdam II clinical diagnostic criteria. We also suggest that screening for ACC should be considered in cancer surveillance strategies directed at individuals with germline mutations in DNA mismatch repair genes.

## Abstract (precis)

This study provides the first description of an intergenerational occurrence of ACC in a family with Lynch Syndrome resulting from a germline MSH2 mutation. Thus, ACC should be included in clinical diagnostic criteria for LS and considered in cancer surveillance recommendations for individuals with germline mutations in DNA mismatch repair genes.

Adrenocortical carcinoma (ACC) is a rare and aggressive endocrine cancer with an incidence of less than 1 case per million individuals per year (1). Most childhood ACC occurs in patients with familial cancer susceptibility syndromes such as Li-Fraumeni syndrome, but whether a hereditary tumor predisposition exists for the development of ACC in adults is less clear (1). Although most ACC in adulthood is sporadic, increasing evidence supports association between adult ACC and inherited cancer susceptibility syndromes including Li-Fraumeni syndrome, multiple endocrine neoplasia type 1, and Lynch syndrome (LS) (1). However, given the low prevalence of ACC, ascertaining whether this cancer is a bona fide syndrome-associated malignancy is challenging.

LS is an autosomal-dominant familial cancer syndrome caused by pathogenic germline mutations in one of several DNA mismatch repair (MMR) genes (*MLH1*, *MSH2*, *MSH6*, or *PMS2),* and associated with an estimated lifetime colorectal cancer risk of 80% (2). LS is also associated with an increased risk of several extracolonic tumors (endometrial, stomach, small intestinal, hepatobiliary, urinary tract), which are therefore included as part of the Amsterdam II clinical diagnostic criteria for LS (2). In addition to these recognized cancers, previous reports have described a number of rare, nonclassical cancers, including ACC, in patients with LS. Given the low prevalence of ACC, it remains uncertain whether it is a true LS-associated tumor or arises independent of the primary genetic defect, although a recent study involving 114 subjects with primary ACC and 135 probands from MMR gene-positive kindreds has provided important evidence for the former (3).

Here, we provide additional evidence to implicate ACC as an LS-associated cancer with the first description of an intergenerational (mother-to-daughter) occurrence of ACC in a family with LS resulting from a germline MSH2 mutation. We therefore propose that ACC be included in clinical diagnostic criteria for LS and considered in cancer surveillance recommendations for individuals with germline mutations in DNA MMR genes.

## Case History

In 2001, the proband (patient III:2), a 54-year old female presented to her local hospital with right loin pain, lethargy, and weight loss. She had previously undergone a hysterectomy and bilateral salpingo-oophorectomy for ovarian cancer at age 44 years and endoscopic removal of a malignant colonic polyp at age 47 years. Clinical examination revealed right loin tenderness but was otherwise unremarkable, with no evidence of catecholamine, glucocorticoid, mineralocorticoid, or androgen excess. Abdominal ultrasonography demonstrated a right suprarenal lesion and subsequent computed tomography confirmed the presence of a 14-cm mass arising from the right adrenal gland. There was no evidence of extraadrenal disease. Biochemical investigations excluded phaeochromocytoma and confirmed a nonsecretory adrenal lesion.

The patient underwent a laparoscopic right adrenalectomy and nephrectomy to remove a 14 × 10 cm adrenal mass. Pathological examination of the resected specimen showed an adrenocortical tumor with adrenal capsular invasion, areas of confluent necrosis, and possible vascular invasion (Figure 2A, a and b). The tumor was composed of lobules of oncocytic cells separated by fibrous septae. The tumor cells showed focal marked nuclear pleomorphism with bizarre nuclear forms. The mitotic index was 1/50 hpf. Immunohistochemically, tumor cells were positive for vimentin and Melan A and negative for calretinin, inhibin, S100, cytokeratin, carcinoembryonic antigen, chromogranin A, neurofilament, and synaptophysin. Collectively, these features were in keeping with an oncocytic AAC based on Lin-Weiss-Bisceglia criteria (4, 5).

Adjunctive mitotane therapy (maximum tolerated dose, 500 mg three times daily) was commenced with concurrent hydrocortisone replacement (10 mg twice daily). To date, the patient has received regular clinical, biochemical, and radiological surveillance with no evidence of disease recurrence.

The proband's sister (patient III:1) had no significant personal medical history until age 36 years when she was diagnosed with an adenocarcinoma of the descending colon, which required a left hemicolectomy. At age 47 years, she was diagnosed with endometrial carcinoma following investigations for intermenstrual bleeding, and treated by radical hysterectomy.

**Figure 1.**
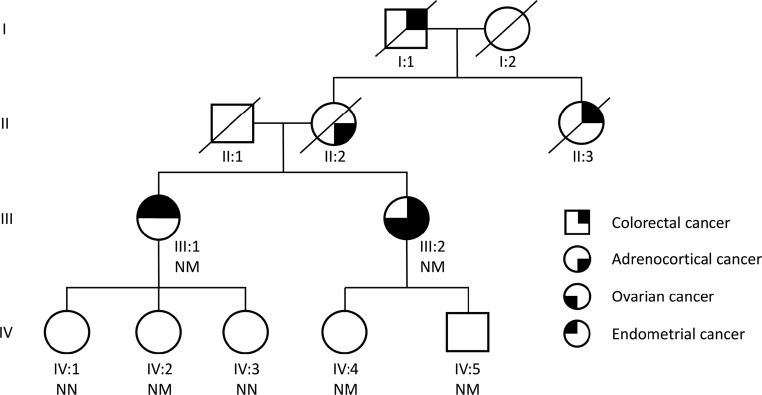
Pedigree of family with the germline *MSH2* mutation. NM, carriers of the mutation; NN, wild-type individuals.

**Figure 2.**
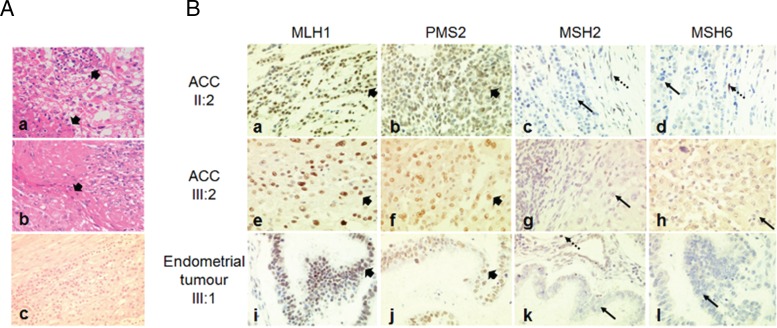
A, a) High-magnification image of hematoxylin and eosin-stained photomicrograph demonstrating confluent tumor necrosis (arrowhead) in ACC from patient II:2. (A, b) Medium-magnification image demonstrating capsular invasion (arrowhead) in ACC from patient II:2. (A, c) Medium-magnification image of ACC from patient III:2 demonstrating atypical nuclei, mitotic activity, and areas of necrosis. (B) Immunohistochemical analyses of MLH1, PMS2, MSH2, and MSH6 protein expression in ACCs resected from patients II:2 (B, a–d) and III:2 (B, e–h), and endometrial tumor resected from patient III:1 (B, i–l) (×400). All analyzed tumors retained expression of MLH1 (B, a, e, i) and PMS2 (B, b, f, j) (short arrowhead), but expression of MSH2 (B, c, g, k) and MSH6 (B, d, h, l) was absent (solid arrow), with adjacent normal stromal cells exhibiting positive staining (dashed arrow).

An underlying familial cancer syndrome was suspected and a more detailed family history revealed that the proband's maternal aunt (patient II:3) and grandfather (patient I:1) had both been diagnosed with colorectal cancer (Figure 1). Importantly, the certificate of death for the proband's mother (patient II:2) stated metastatic ACC as the cause of death. Given the rarity and difficulty in establishing malignancy of adrenocortical tumors, the histology of the resected adrenal tumor was reevaluated and confirmed the original diagnosis of primary AAC (Figure 2A, c).

In accordance with the Amsterdam II criteria and revised Bethesda criteria, the family fulfilled diagnostic requirements for LS and further immunohistochemistry was performed on both ACCs (patients III:2 and II:2) and the endometrial tumor (patient III:1) to determine MMR protein expression status (Figure 2B) (2). In all analyzed tumors, expression of MLH1 and PMS2 was retained, whereas nuclear staining for MSH2 and MSH6 were absent, which is consistent with a LS phenotype. Further germline genetic testing was undertaken by multiplex ligation dependent probe amplification analysis in the proband and her sister and revealed a heterozygous deletion of exons 1, 2, and 3 of the *MSH2* gene. Further genetic testing also identified the mutation in the proband's son (patient IV:5), daughter (patient IV:4), and niece (patient IV:2). In accordance with the Chompret testing criteria, *TP53* germline mutations were excluded in the proband (6). The unavailability of genomic DNA from the proband's mother (patient II:2) precluded further genetic study.

## Discussion

ACC is not currently included in the diagnostic criteria for LS. However, our description of familial ACC arising in the context of a germline *MSH2* mutation supports the recent proposal that ACC should be considered an LS-associated tumor (3). Currently, the diagnosis of LS requires patients/kindreds to fulfill the Amsterdam or revised Bethesda criteria, with demonstration of absent MMR protein expression on tumor immunohistochemistry and/or microsatellite instability (MSI) genotyping supporting the diagnosis (2). Genetic testing for germline mutations in MMR genes is reserved for individuals with tumors that demonstrate MSI or absent MMR protein expression, or those deemed at risk based on computational prediction models (2).

Isolated cases of ACC arising in single patients of families with LS and germline mutations in DNA MMR genes (*MLH1, MSH2, MSH6*) have been previously reported, but given their rarity, determining whether these tumors were coincidental or part of the LS tumor profile has remained controversial (3, 7, 8). Compelling evidence supporting ACC as an LS-associated cancer was recently provided by Raymond and colleagues, who found that LS prevalence among patients with primary ACC was significantly higher (3.2%) than in the background population (0.2%), and comparable to the prevalence of LS in colorectal (2–4%) and endometrial cancer (1–5%) (3). Moreover, the prevalence of ACC in LS was increased compared with the general population. Given that ACC is typically an aggressive malignancy, with limited treatment options for advanced disease, recognizing association of this tumor with LS has important clinical implications. Specifically, improved awareness and recognition of the syndrome and entry into appropriate systematic cancer surveillance programs would be anticipated to lead to earlier diagnosis and timely intervention to reduce LS-related morbidity and mortality.

Immunohistochemical (IHC) analysis of tumor MMR proteins is often performed concomitantly with MSI to improve clinical sensitivity in the evaluation for LS. In our case, IHC analysis for LS-related tumors was informative and identified loss of MSH2 and MSH6 expression in the ACC and endometrial tumors analyzed from affected patients with the germline MSH2 mutation. In contrast with colorectal tumors, which demonstrate high MSI, previous reports have consistently found that ACC tumors have low MSI in patients with LS; on this basis, MSI testing was not performed in our patient (3). Although a molecular basis for this discrepancy between tumor types remains unclear, it has been proposed that in some tumors, possibly from tissue-specific factors, the consequences of MMR deficiency occur in the latter stages of carcinogenesis thereby preventing detectable accumulation of MSI. Further, currently used MSI tests have been optimized for colorectal cancers and are less sensitive for MSI detection in other tumor types. Therefore, low MSI does not necessarily exclude a diagnosis of LS and, for both sporadic and familial ACC, IHC analysis of MMR proteins should be considered the first-line molecular screening strategy, even in individuals without other LS-associated lesions, with germline genetic testing pursued in the absence of one or more MMR proteins. Currently, there are no established biochemical or radiological screening guidelines for those at risk of ACC. We have adopted an empirical approach in the offspring of our proband, combining periodic surveillance magnetic resonance imaging with serum and urinary steroid profiling. It remains to be seen whether this is a clinically cost-effective approach.

In summary, we report the first description of familial ACC in conjunction with a germline *MSH2* mutation and provide support for MMR genes as candidates in hereditary ACC. We advocate ACC now be included in clinical diagnostic criteria for LS and considered in cancer surveillance strategies for individuals with germline mutations in DNA MMR genes. Moreover, in the absence of clinical management guidelines for ACC surveillance in patients with inherited cancer syndromes, including LS, we recommend individualized screening protocols coupled with ongoing clinical vigilance.
